# Conversion of rice husks into carbonaceous materials with porous structures via hydrothermal process

**DOI:** 10.1007/s11356-024-34217-6

**Published:** 2024-07-08

**Authors:** Sayaka Sugie, Hirotaka Maeda

**Affiliations:** Department of Life Science and Applied Chemistry, Nagoya Institute of Technology, Gokiso-Cho, Showa-Ku, Nagoya, 466-8555 Japan

**Keywords:** Rice husk, Hydrothermal process, Porosity properties, Acidic solvent, Carbonaceous material, Amorphous

## Abstract

Carbonaceous materials hydrothermally produced using waste biomass have small specific surface areas (SSA) and poor porosity properties. In this study, we prepare a novel carbonaceous material with excellent porosity properties by suppressing the formation of a secondary char phase (spheres) and promoting biomass hydrolysis by controlling the hydrothermal conditions. Rice husk powders, as the starting material, are hydrothermally treated using acidic solvents of different types and concentrations at 180 °C. The surfaces of the samples hydrothermally prepared using the acidic solvents have no spheres. In the case of 0.1–0.2 mol L^−1^ hydrochloric acid (HA), the amorphous carbonaceous materials contain numerous mesopores and exhibit a larger SSA (approximately 100 m^2^ g^−1^) than those prepared using acetic acid and distilled water. An increase in the hydrothermal temperature reduces the porosity properties of the materials. Finally, the high-porosity amorphous carbonaceous material showed excellent trimethylamine adsorption ability.

**Supplementary Information:**

The online version contains supplementary material available at 10.1007/s11356-024-34217-6.

## Introduction

In recent years, various groups have studied the use of biomass as a starting material to prepare functional materials for sustainable societies (Sri et al. 2021). Biomass is converted into accessible resources through thermochemical processes, such as pyrolysis and hydrothermal carbonization. Pyrolysis is performed at reaction temperatures exceeding 300 °C in the absence of oxygen. Hydrothermal carbonization is an environmentally friendly method that converts wet biomass into carbonaceous materials with surface functional groups at a self-generated pressure at temperatures of 180–250 °C (Masoumi et al. 2021; Shen 2020). Hydrothermally carbonized biomass, termed hydrochar, is used in various applications, such as adsorption, catalysis, and supercapacitors (Chhabra et al. 2022; Ghanim et al. 2022; Liu et al. 2020).

Hydrochar consists of a primary char surface, which originates from the solid–solid carbonization reactions in the biomass, and a secondary char phase, which consists of spheres formed via polymerization and condensation reactions by the solid–liquid interfaces on the primary char surface (Karayildirim et al. 2008; Volpe and Fiori 2017). Hydrochar lacks porosity and has a small surface area, mainly due to the presence of interparticle voids (Zhu et al. 2015). Researchers have attempted to address this issue by synthesizing activated carbon with good porosity properties via the heat treatment of hydrochar (Huang et al. 2020; Shen et al. 2021; Zhang et al. 2019).

Rice husk–derived hydrochar was prepared using microwaves; it had a specific surface area (SSA) of more than 90 m^2^ g^−1^, and its surface had some pores but no spheres (Nizamuddin et al. 2018). Compared with its pretreatment state, hydrochar derived from *Eupatorium adenophorum* using mild hydrothermal carbonization had a rougher surface and a more porous structure without spherical deposits (Han et al. 2023). Thus, suppressing the formation of the secondary char phase (as spheres) may be important for converting biomass into high-porosity carbonaceous materials using the hydrothermal process. Our novel preparation strategy promotes the decomposition of biomass components, such as hemicellulose and cellulose, and inhibits polymerization and condensation reactions by controlling the hydrothermal conditions.

During hydrothermal carbonization, polymerization and recombination reactions possibly begin at approximately 180–185 °C, whereas hydrolysis is dominant at relatively low temperatures (under 220 °C) (Djandja et al. 2023). In a study on the hydrothermal carbonization of cellulose at 210–250 °C, samples prepared at over 220 °C consisted mainly of microsphere aggregates (Sevilla and Fuertes 2009). Hence, conducting the hydrothermal process at 180 °C may suppress the formation of spheres and promote the hydrolysis of biomass as the starting material. Compared with the hydrothermal carbonization of wheat straw in a basic medium, that in an acidic medium resulted in a larger surface area and a higher pore volume (Reza et al. 2015). Hydrochar prepared using citric acid as the solvent contained newly formed mesopores, depending on the type of biomass used as the starting material (Titirici et al. 2007). The addition of acids, such as diluted hydrochloric acid (HA), sulfuric acid, and nitric acid, significantly influences the hydrothermal reaction pathway at 220–260 °C (Zhang et al. 2021). Therefore, an acidic solution is a suitable solvent in the hydrothermal process. In this study, different types and concentrations of acidic solvents under hydrothermal conditions at 180 °C were explored to prepare novel carbonaceous materials with excellent porosity properties from waste biomass.

## Material and methods

Rice husks were used as waste biomass. Raw husks of Japanese Hatsushimo rice were collected from Gifu Prefecture. They were washed with distilled water to remove surface impurities and dirt and then dried in an oven at 60 °C for 12 h. The dried rice husks were pulverized and sieved through a 300 μm mesh; the resulting powder was the starting material. The X-ray diffraction (XRD) pattern, scanning electron microscopy (SEM) image, and nitrogen gas adsorption isotherm of the rice husk powder are shown in Fig. SI-1. The SSA of the powder was determined to be 3 m^2^ g^−1^ using the Brunauer–Emmett–Teller method.

Acid enhances biomass dehydration and releases hydrogen ions in the hydrothermal process. In this study, diluted HA and acetic acid (AA) were used as acidic solvents to clarify the influence of the difference between organic and inorganic acids during the hydrothermal process on the preparation of porous carbonaceous materials. For comparison, distilled water was also used. A slurry with a solid/solvent ratio (weight ratio) of 1/10 was prepared by placing 1.5 g of the rice husk powder and 15 g of the solvent in a 50-mL Teflon container. The slurry was hydrothermally reacted at 180 °C for 20 h at 50 rpm. The reaction time was determined through optimization based on a trial-and-error approach to achieve samples with satisfactory SSAs in our preliminary experiments. After the hydrothermal process, the samples were washed with distilled water, separated through filtration, and dried at 60 °C for 12 h. The resulting samples were stored in vacuum desiccators until further experimentation. The samples prepared using different solvents were labeled using the acid concentration and the solvent name abbreviation; for example, 0.1HA was prepared using 0.1 mol L^−1^ HA, and DW was prepared using distilled water.

The crystallinity of the samples was determined through XRD with CuKα radiation (*λ* = 0.15418 nm) at 40 mA and 45 kV at a scanning rate of 0.04°/s. Their morphologies were characterized via SEM. The nitrogen gas adsorption isotherms of the samples were determined at − 196 °C through nitrogen gas adsorption analysis. The samples were degassed in a vacuum at 100 °C for 3 h. The carbon, hydrogen, nitrogen, and sulfur contents of the samples were determined using an elemental analyzer. The ash content of each sample was calculated from its weight loss after being heated at 1000 °C for 1 h. The oxygen content was calculated using Eq. (1) (Kang et al. 2012).

1 $$O=100-\left(C+H+N+S+Ash\right)$$

## Result and discussion

**Fig. 1:**
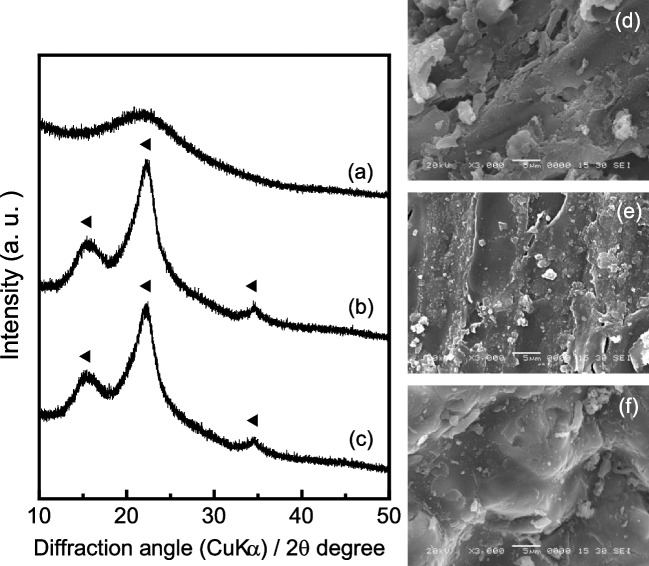
XRD patterns and SEM images of samples prepared using different solvents. (**a**, **d**) 0.1HA (**b**, **e**) 0.1AA, and (**c**, **f**) DW. (◂) Cellulose

Figure 1 shows the XRD patterns and SEM images of the samples prepared using distilled water and the acidic solvents at 0.1 mol L^−1^. Peaks similar to those of the rice husk powder are observed at approximately 16°, 22°, and 34° in the XRD patterns of 0.1AA and DW (Fig. SI-1). Hydrochar prepared from orange peels using 3.3 × 10^−3^ mol L^−1^ citric acid at 200 °C for 24 h exhibited amorphous phases (Chhabra et al. 2022). The transformation of the crystalline cellulose in the biomass into these amorphous phases was influenced by the raw materials and reaction temperature. In the case of 0.1HA, a halo peak is observed at approximately 21° without crystalline cellulose peaks, which implies the presence of an amorphous structure. These results suggest that the hydrolysis of crystalline cellulose in AA under the experimental conditions is insufficient compared with that in HA.

The morphologies of 0.1AA and DW are similar to that of the rice husk powder. On the contrary, 0.1HA has a rougher surface than the rice husk powder; it also has some pores without spherical deposits (Fig. 1(d)). The production yield without an acidic solvent is 62.0%, whereas those in the presence of HA and AA are 44.0% and 63.6%, respectively. The hydrochar yields obtained from rice husks using deionized water at 200 °C for 30 min and 4 h were approximately 72% and 74%, respectively (Nakason et al. 2018; Raheem et al. 2022). Thus, the hydrothermal process in HA at 180 °C may promote cellulose hydrolysis while suppressing polymerization and condensation, leading to the formation of amorphous carbonaceous materials.

**Fig. 2:**
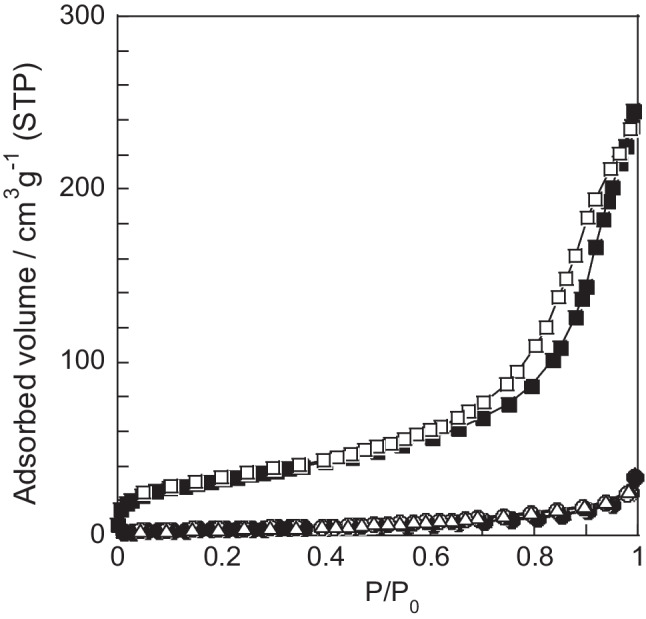
Nitrogen gas adsorption isotherms of samples prepared using different solvents. (square) 0.1HA, (triangle) 0.1AA, and (circle) DW. The filled and unfilled marks represent adsorption and desorption branches, respectively

Figure 2 shows the nitrogen gas adsorption isotherms of the samples prepared using distilled water and the acidic solvents at a concentration of 0.1 mol L^−1^. Notably, 0.1AA and DW exhibit slightly higher nitrogen gas adsorption capacities than the rice husk powder. By contrast, 0.1HA exhibits type IV isotherms with H3 hysteresis loops (IUPAC classification). In addition, 0.1HA adsorbs approximately 25 times more nitrogen gas than the rice husk powder. The sample prepared using distilled water has an SSA of 13 m^2^ g^−1^ and a pore volume of 0.03 cm^3^ g^−1^. The SSA and pore volume of 0.1AA are 14 m^2^ g^−1^ and 0.03 cm^3^ g^−1^, respectively, which are slightly higher than those of the rice husk powder. Furthermore, 0.1HA has an SSA of 118 m^2^ g^−1^ and a pore volume of 0.33 cm^3^ g^−1^. The rice husk–derived hydrochar prepared using distilled water at 180 °C for 8 h and at 200 °C for 16 h exhibits SSAs of 20 and 30 m^2^ g^−1^, respectively (Kalderis et al. 2014; Zhang et al. 2022). Rice husk–derived hydrochar samples prepared through metal chloride–assisted hydrothermal carbonization had SSAs of 13–45 m^2^ g^−1^ (Li et al. 2021). Pine cone–derived hydrochar prepared using citric acid at a concentration of approximately 1.3 × 10^−3^ mol L^−1^ at 200 °C for 16 h exhibited an SSA of 34 m^2^ g^−1^, and a hysteresis loop was observed in its nitrogen isotherm (Titirici et al. 2007). Therefore, conducting the hydrothermal process using HA at 180 °C plays an important role in increasing both the SSA and pore volume of carbonaceous materials.

Samples hydrothermally prepared using 0.1 mol L^−1^ HA for 5 h (0.1HA-5h) show a 35 m^2^ g^−1^ SSA and a 0.04 cm^3^ g^−1^ pore volume. The SSA and pore volume of 0.1HA are 3.4 and 8.3 times higher, respectively, than those of 0.1HA-5h. The hydrochar hydrothermally treated in 5 mol L^−1^ HA for 12 h shows a 22 m^2^ g^−1^ SSA and a 0.08 cm^3^ g^−1^ pore volume (Luo et al. 2024). These suggest that a prolonged hydrothermal time is important for improving the SSA and porosity properties of carbonaceous materials. The porosity properties do not significantly change when the hydrothermal time is further extended. For example, the sample hydrothermally treated with 0.1 mol L^−1^ HA for 70 h has a 108 m^2^ g^−1^ SSA and a 0.34 cm^3^ g^−1^ pore volume.

**Fig. 3:**
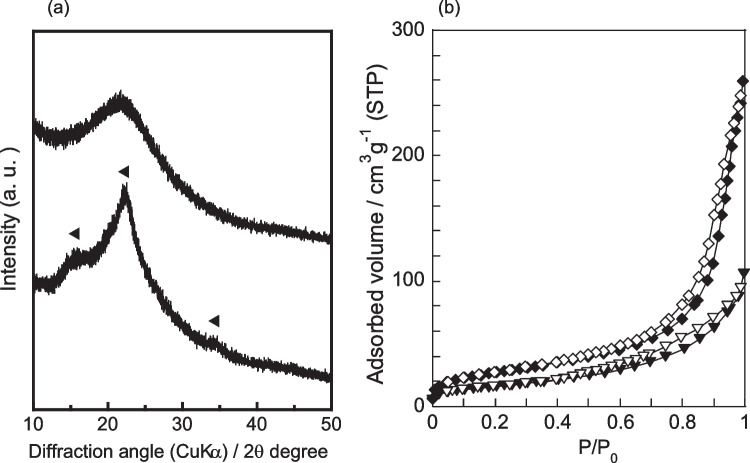
(**a**) XRD patterns and (**b**) nitrogen gas adsorption isotherms of samples prepared using different HA concentrations. (Lower pattern in XRD, reverse triangle in isotherm) 0.05HA and (upper pattern in XRD, diamond in isotherm) 0.2HA. The filled and unfilled marks represent adsorption and desorption branches, respectively. (◂) Cellulose

The effects of the HA solvent concentration on sample porosity were investigated. Figure 3 shows the XRD patterns and nitrogen gas adsorption isotherms of the samples prepared using different concentrations of HA. Crystalline cellulose peaks are observed in the XRD pattern of 0.05HA. By contrast, in the XRD pattern of 0.2HA, a halo peak due to the amorphous structure is observed at 21°, which is almost the same as the case of 0.1HA. Thus, the crystallinity of cellulose in the samples depends on the solvent concentration. The SSA and pore volume of 0.05HA are 57 m^2^ g^−1^ and 0.12 cm^3^ g^−1^, respectively. Similar to 0.1HA, 0.2HA has a 98 m^2^ g^−1^ SSA and a 0.35 cm^3^ g^−1^ pore volume. The sample prepared using 0.4 mol L^−1^ HA has almost the same SSA (98 m^2^ g^−1^) and pore volume (0.34 cm^3^ g^−1^). Therefore, increasing the solvent concentration beyond 0.1 mol L^−1^ has negligible effects on improving the SSA and pore volume of the sample under this experimental condition. Samples 0.05HA and 0.2HA have similar morphologies (Fig. SI-2). The porosity properties of the samples have to be enhanced to change their morphologies, such as surface roughening and lowering cellulose crystallinity.

**Fig. 4:**
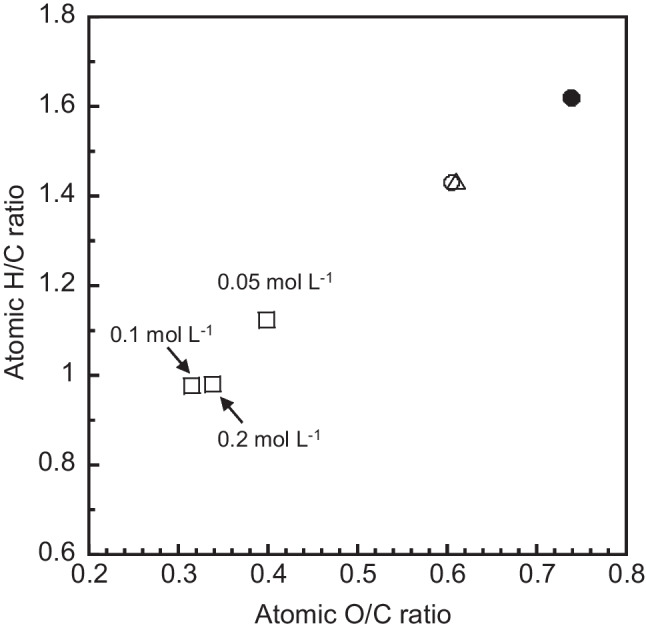
van Krevelen diagram of samples. (●) Rice husk powder; samples prepared using (□) HA: 0.05–0.2 mol L^−1^, (△) AA: 0.1 mol L^−1^, and (◯) DW as solvent

The elemental analysis results are displayed in Fig. 4 as a van Krevelen diagram of the O/C and H/C atomic ratios of all samples and the rice husk powder as the starting material. The O/C and H/C ratios of 0.1AA are 0.61 and 1.43, respectively, which are almost the same as those of DW. Hydrochar derived from rice husks subjected to hydrothermal carbonization at 180 °C for 30 min using deionized water had an O/C ratio of 0.7 (Raheem et al. 2022), which is similar to that of 0.1AA. The O/C ratios of 0.05HA, 0.1HA, and 0.2HA are 0.40, 0.31, and 0.34, respectively. The O/C ratios of the samples prepared using HA are significantly lower than that reported for rice husk–derived hydrochar. The carbon content of the samples increases with the HA concentration, but further increasing the concentration beyond 0.1 mol L^−1^ has little effect on the carbon content. The H/C ratios of 0.05HA, 0.1HA, and 0.2HA, are 1.12, 0.98, and 0.98, respectively. However, increasing the HA concentration beyond 0.1 mol L^−1^ has little effect on the H/C ratio of the samples. Therefore, the degradation reaction pathway, due mainly to the dehydration of the organic components in the rice husks, proceeds more effectively in the presence of HA than in the presence of AA and distilled water, as seen in the van Krevelen diagram.

**Fig. 5:**
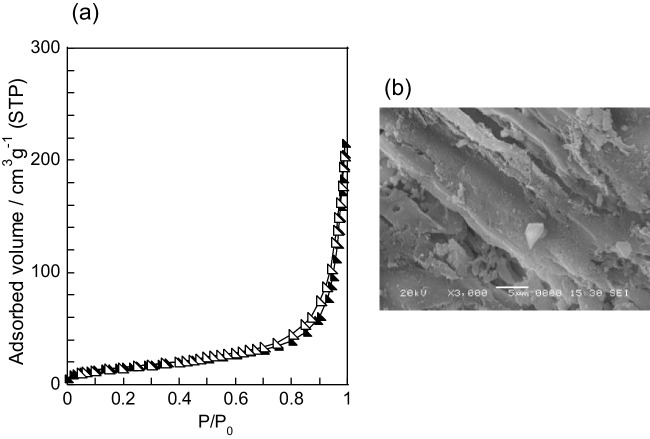
(**a**) Nitrogen gas adsorption isotherm and (**b**) SEM image of sample prepared using 0.1 mol L^−1^ HA at 210 °C. The filled and unfilled marks represent adsorption and desorption branches, respectively

Hemicellulose hydrolyzes at approximately 180 °C under hydrothermal conditions, whereas the hydrothermal degradation of lignin and cellulose is most likely realizable at above 200 °C (Funke and Ziegler 2010). Increasing the dehydration amounts of the organic components in the starting material may improve the porosity properties of samples. A sample was hydrothermally treated using 0.1 mol L^−1^ HA at 210 °C to clarify the effect of the hydrothermal temperature on the porosity properties of the amorphous carbonaceous material. Figure 5 shows the nitrogen gas adsorption isotherm and SEM image of the sample. Its SSA and pore volume are 56 m^2^ g^−1^ and 0.20 cm^3^ g^−1^, respectively. The sample surface has no deposits but has some pores, as shown in Fig. 5(b). The porosity properties are dramatically lower than those of 0.1HA. The temperature increase degrades the porosity properties of the sample. The production yield is 43.7%, which is almost the same as that of 0.1HA. An increase in the reaction temperature reduces the hydrochar yield at 140–300 °C (Ameen et al. 2022). Hence, the reaction temperature of the hydrothermal process using diluted HA has no effect on the conversion yield of rice husks. Carbonaceous materials with unique porous structures derived from waste biomass can be simply prepared using the hydrothermal process at a low temperature using HA of an adequate concentration.

We hypothesized that the introduction of unique porous structures into carbonaceous materials hydrothermally prepared from biomass enhances the adsorption performance of these materials for environmental remediation applications. Therefore, 0.01 g of each sample was placed in a gas bag with 200 ppm of gaseous trimethylamine for 2 h to examine its ability to adsorb trimethylamine as a harmful gas. The trimethylamine concentration was examined using a detecting tube. The results show that 0.1HA has trimethylamine adsorption performance (77 ± 11 ppm). To compare the reported carbon materials with different specific surface area, the adsorbed amounts of trimethylamine were calculated by dividing it by specific surface areas of the samples. 0.1HA has similar adsorption property (0.17 ± 0.02 mg m^−2^) to activated carbon (~ 0.10 mg m^−2^) (Lee et al. 2010). Investigations regarding equilibrium and kinetic theories are ongoing for a more in-depth discussion on the trimethylamine adsorption properties of the samples. The carbon filter fabricated using the carbonized rice husk with 168 m^2^ g^−1^ of SSA, prepared via pyrolysis at 450 °C, did not show any reduction trimethylamine in the adsorption test for 30 min (Nam et al. 2018). For the carbon filter fabricated using activated carbon with and without copper impregnation, trimethylamine adsorption was achieved in an enclosed chamber (Nam et al. 2018; Wang et al. 2019). These imply that using amorphous carbonaceous material in a carbon filter is a promising adsorbent for air environmental remediation.

## Conclusion

This study clarifies the effects of the hydrothermal process for the conversion of rice husk powder into carbonaceous materials on their porosity properties. Amorphous carbonaceous materials hydrothermally prepared using diluted HA at 180 °C for 20 h had a higher SSA and pore volume than those in previous reports on hydrochar derived from rice husks using the hydrothermal process. Surface morphology changes without the formation of spheres and the disappearance of crystalline cellulose as the starting material are key factors in developing the porosity properties of these materials. Excessive increases in the reaction temperature, reaction time, and solvent concentration had negligible effects on the improvement of the porosity properties. Finally, the materials prepared using 0.1 mol L^−1^ HA have excellent trimethylamine adsorption capacity.

### Supplementary Information

Below is the link to the electronic supplementary material.

Supplementary file1 (PPTX 2962 KB)

## Data Availability

The datasets used and/or analyzed during the current study are available from the corresponding author on reasonable request.
