# Supplementary material for: Conversion of rice husks into carbonaceous materials with porous structures via hydrothermal process
Source: Environ Sci Pollut Res Int. 2024 Jul 8;31(33):45711–7. doi: 10.1007/s11356-024-34217-6 (PMC11269380; doi:10.1007/s11356-024-34217-6)
Supplement: Supplementary file 1 — Supplementary file1 (PPTX 2962 KB) [file 11356_2024_34217_MOESM1_ESM.pptx]

## Slide 1
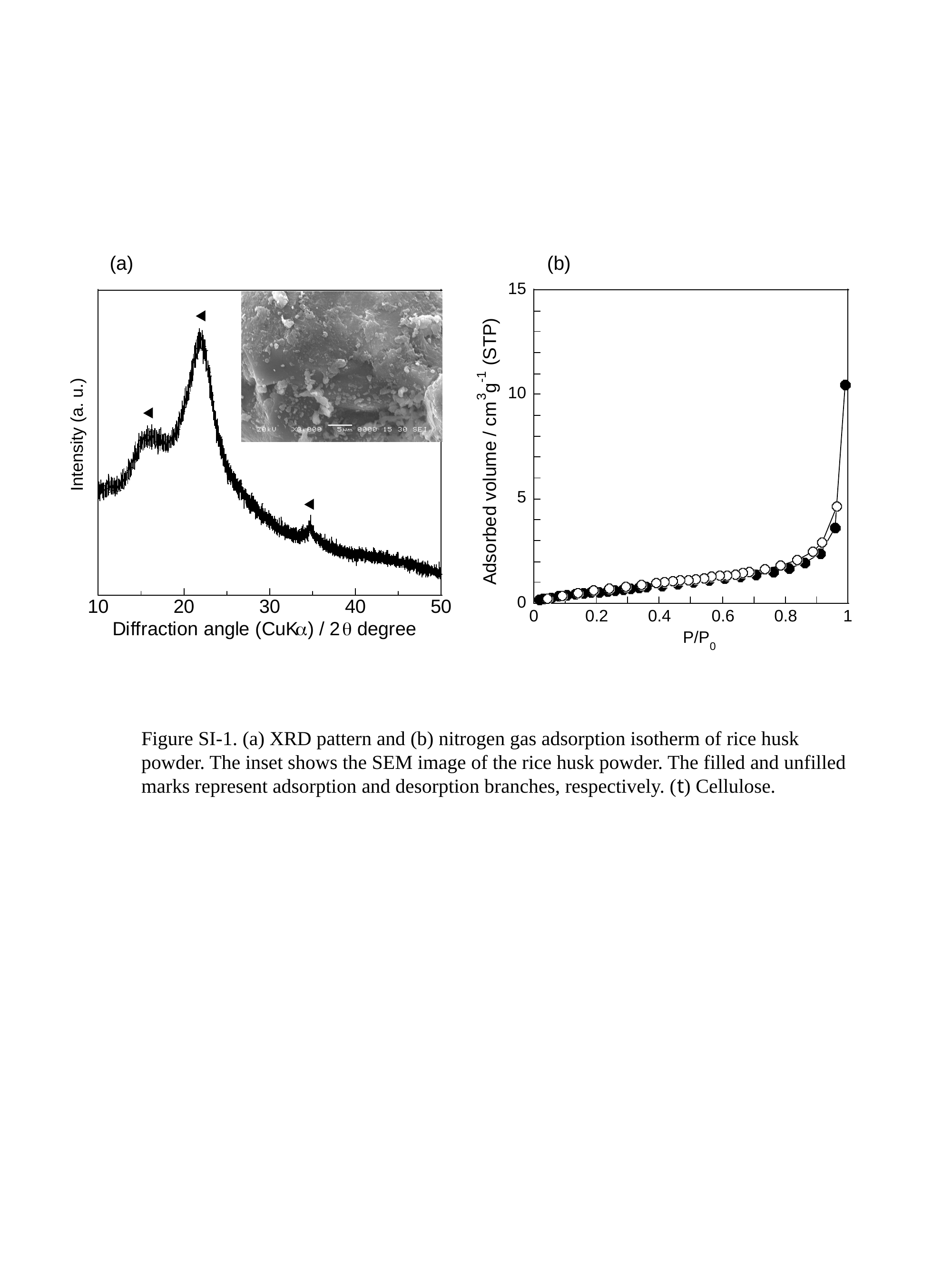

(a)
(b)
Figure SI-1. (a) XRD pattern and (b) nitrogen gas adsorption isotherm of rice husk powder. The inset shows the SEM image of the rice husk powder. The filled and unfilled marks represent adsorption and desorption branches, respectively. (t) Cellulose.

## Slide 2
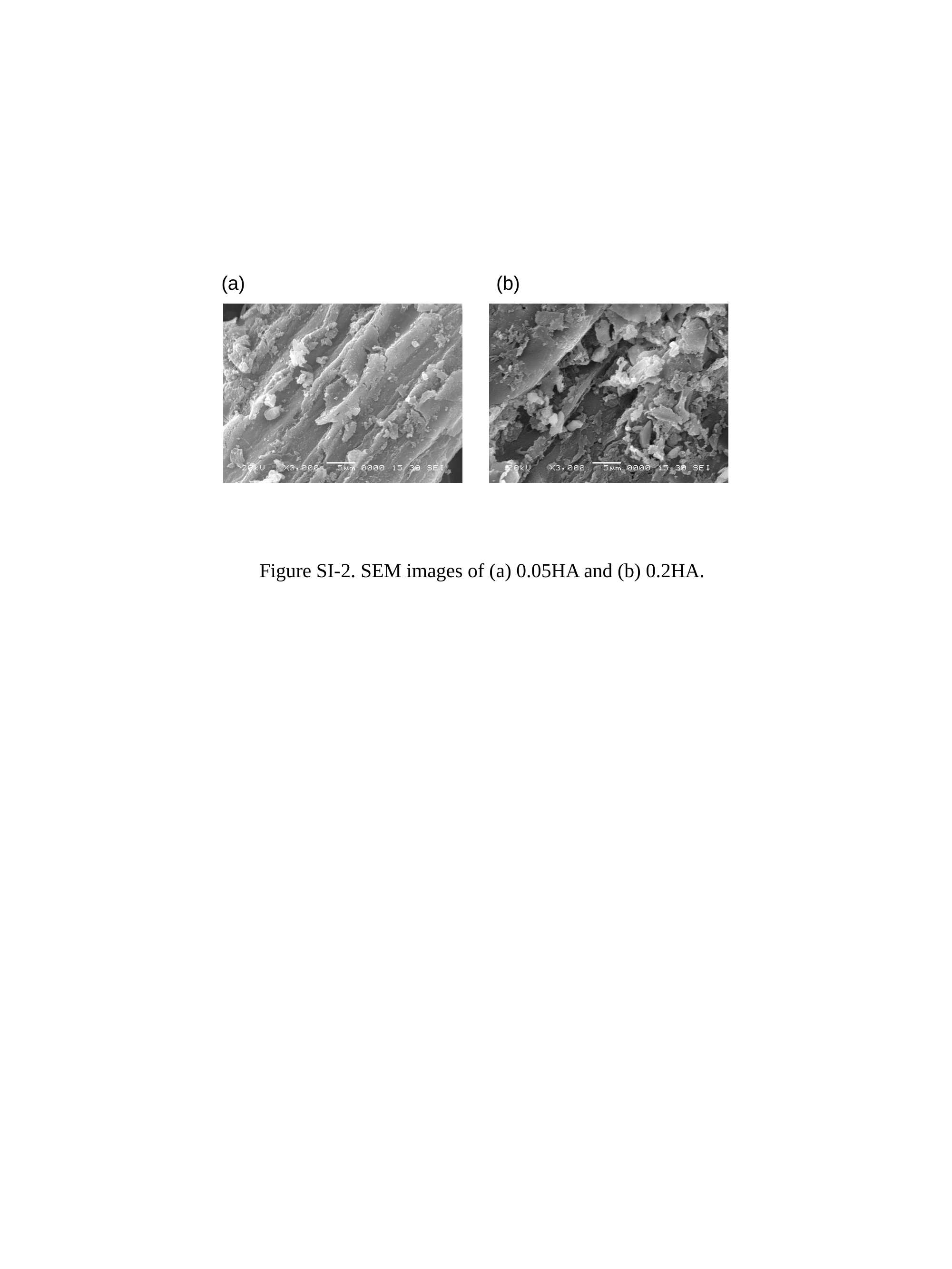

(a)
(b)
Figure SI-2. SEM images of (a) 0.05HA and (b) 0.2HA.
